# Network pharmacology and bioinformatics insight into the mechanism of GeGen-QinLian decoction in colorectal cancer and type 2 diabetes mellitus

**DOI:** 10.1097/MD.0000000000043274

**Published:** 2025-07-18

**Authors:** Jinhao Liang, Chengjiang Xiang, Yuanxiao Liang

**Affiliations:** a Department of Anorectal Surgery, Shengzhou People’s Hospital (the First Affiliated Hospital of Zhejiang University Shengzhou Branch), Shenzhou, China; b Department of Anorectal Surgery, Xinchang County People’s Hospital, Shaoxing, Zhejiang Province, China.

**Keywords:** colorectal cancer, GeGen-QinLian decoction, immune infiltration, network pharmacology, type 2 diabetes mellitus

## Abstract

Colorectal cancer (CRC) and type 2 diabetes mellitus (T2DM) exhibit interrelated pathologies, yet the underlying mechanisms of their interaction remain largely elusive. GeGen-QinLian decoction (GQD) has shown therapeutic efficacy in both CRC and T2DM. This study aimed to elucidate the potential pharmacological mechanisms of GQD in the postoperative treatment of patients with CRC and T2DM. Transcriptomic data sets for CRC and T2DM were retrieved from The Cancer Genome Atlas and Gene Expression Omnibus databases. Differential expression analysis, univariate Cox regression analysis, and weighted gene coexpression network analysis were employed to identify shared genes between CRC and T2DM. Network pharmacology was used to analyze the bioactive components of GQD and their targets, identifying potential therapeutic targets for the concurrent treatment of T2DM and CRC. Enrichment analysis, immune infiltration assessment, and drug sensitivity analysis were performed, complemented by molecular docking to validate the affinity between potential targets and active components. A total of 433 shared genes between CRC and T2DM were identified, involving processes such as gene expression regulation, cell cycle control, apoptosis regulation, Wnt signaling pathway, regulation of NF-κB transcription factor activity, and inflammatory mediator regulation of transient receptor potential channels. We identified 204 bioactive components of GQD and 320 corresponding targets, of which 10 (ADRA1B [adrenergic receptor alpha 1B], CALM1 [calmodulin 1], CDKN2A [cyclin-dependent kinase inhibitor 2A], CTNNA1 [cadherin-associated protein], FCER2 [Fc fragment of IgE receptor II], GSR [glutathione reductase], GSTM1 [glutathione S-transferase mu 1], IL13 [interleukin 13], INSR [insulin receptor], and MAPK9 [mitogen-activated protein kinase 9]) were determined as potential targets for the treatment of T2DM and CRC using GQD. Enrichment analysis revealed that these targets were associated with pathways including insulin signaling pathway, cyclic guanosine monophosphate-protein kinase G signaling pathway, Ras signaling pathway, and Fc-epsilon receptor I signaling pathway. Molecular docking results demonstrated high affinity between these potential targets and active components, with the highest affinity observed between CALM1 and xambioona. This study systematically identified a set of shared genes between T2DM and CRC, along with the bioactive components and 10 potential targets of GQD for the treatment of T2DM and CRC. These findings provided a theoretical foundation for the combined therapy of T2DM and CRC.

## 1. Introduction

Colorectal cancer (CRC) ranks as the third most common cancer in the United States, with an estimated 152,810 new cases and 53,010 deaths projected for 2024.^[1]^ The number of patients with CRC in China continues to rise, with the incidence of male CRC ranking fourth among all malignant tumors and female third.^[2]^ Diabetes has been considered as an independent risk factor for CRC, especially type 2 diabetes mellitus (T2DM). As of 2021, the number of diabetes patients reached about 529 million worldwide and 117 million in China, with projections suggesting this figure could surge to around 1.31 billion globally by 2050.^[3–5]^ The presence of diabetes significantly impacts the quality of life of patients following radical resection of CRC. Therefore, effective perioperative blood glucose management is essential to reduce surgical risks and enhance postoperative quality of life in patients with CRC and diabetes.

Gegen-Qinlian decoction (GQD), originates from Treatise on Febrile Diseases, is a traditional Chinese herbal formula comprising 4 medicinal herbs (Table 1). Clinical applications have demonstrated that GQD is effective in treating both CRC and T2DM. For instance, Li et al.^[6]^ found that the symptoms of patients with CRC, such as abdominal pain, diarrhea, and tenesthesia, were significantly improved after GQD treatment. Similarly, Lv et al.^[7]^ found that GQD treatment could significantly decrease the tumor size of mice with CRC, indicating that GQD could inhibit the growth of CRC tumor cells. A meta-analysis further confirmed the favorable therapeutic effect of GQD on T2DM,^[8,9]^ and Xu et al.^[10]^ demonstrated its notable hypoglycemic effects on diabetic rats. Despite these findings, the potential value and pharmacological mechanism of GQD in the treatment of CRC and T2DM remain underexplored.

**Table 1:** The detailed information of the 4 kinds of herbal medicines that make up GQD.

Chinese name	Species	Part
Gegen	*Pueraria lobata* (Willd.) Ohwi or *Pueraria thomsonii* Benth.	Root
Huangqin	*Scutellaria baicalensis* Georgi	Root
Huanglian	*Coptis chinensis* Franch.	Root or stems
Gancao	*Glycyrrhiza uralensis* Fisch or *Glycyrrhiza inflata* Bat. Or *Glycyrrhiza glabra* L.	Root or stems

GQD = GeGen-QinLian decoction.

Traditional Chinese medicine (TCM) is a complex system with multiple targets and synergistic or antagonistic interactions among its components, which makes conventional pharmacological approaches not applicable to TCM research. Network pharmacology, an emerging field, offers a comprehensive and dynamic approach to analyzing disease, gene, protein target and drug interactions. This method can effectively elucidate the pharmacological mechanism of TCM at a holistic level. Over the past decades, network pharmacology has been widely and successfully applied to investigate the pharmacological mechanism of various TCMs.^[11]^

**Figure 1.**
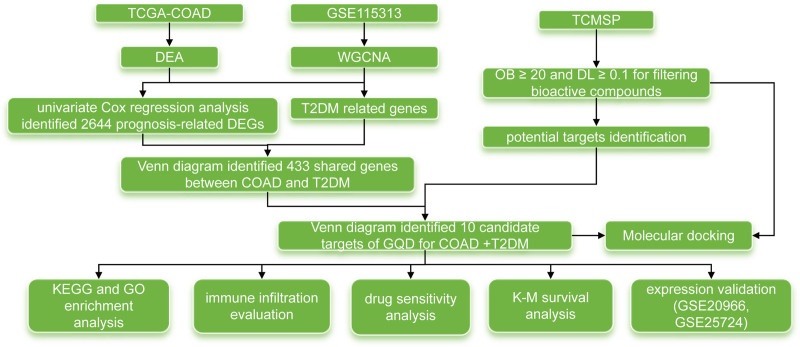
The workflow diagram of this study.

It is widely recognized that exploring common genetic features between CRC and T2DM is of significant importance for developing new strategies for joint prediction, prevention, and intervention. In this study, we utilized bioinformatics to investigate the shared genetic characteristics between T2DM and CRC. We then integrated network pharmacology and molecular docking analysis to elucidate the underlying mechanism of GQD in the treatment of CRC and T2DM. The workflow of this study is illustrated in Figure 1.

## 2. Materials and methods

### 2.1. Data acquisition and processing

Transcriptomic data sets related to T2DM were downloaded from the Gene Expression Omnibus database (https://www.ncbi.nlm.nih.gov/geo/). The data sets included: GSE115313 (tumor samples and normal colonic mucosa from 23 nondiabetic and 19 diabetic patients with colon cancer), GSE20966 (transcriptomic profiles from pancreatic β cells of 10 control and 10 T2DM patients), and GSE25724 (expression data from type 2 diabetic and nondiabetic isolated human islets). Transcriptional and clinical information from the colorectal adenocarcinoma (COAD) cohort was obtained from The Cancer Genome Atlas (TCGA, https://portal.gdc.cancer.gov/), which includes transcriptomic data from 41 normal tissues and 473 colorectal cancer tissues. All transcriptomic data were normalized using the normalizeBetweenArrays function from the limma package and log2-transformed.

### 2.2. Identification of T2DM-related genes

Using the GSE115313 data set, weighted gene coexpression network analysis (WGCNA) and differential expression analysis were conducted to identify genes associated with T2DM. WGCNA was performed using the WGCNA package.^[12]^ Outliers were removed by constructing a sample clustering tree based on cutHeight. A soft threshold power β was defined to ensure the scale-free topology of the network. Adjacency matrices were transformed into topological overlap matrices, and different gene modules were identified using the dynamic tree-cutting algorithm. Modules significantly correlated with T2DM were selected. Differential expression analysis was conducted using the limma package, with a *P*-value threshold of <0.05. Volcano plots were generated using the ggplot2 package in R. The union of genes identified through WGCNA and differential expression analysis was considered as T2DM-related genes.

### 2.3. Identification of CRC-related genes

Differential expression analysis of genes between tumor and adjacent normal tissues in the TCGA-COAD cohort was performed using the limma package, with adjusted *P*-value thresholds of < .05 and log2(fold change) > 1. Univariate Cox regression analysis was used to determine the prognostic relevance of differentially expressed genes in colorectal cancer. Genes with *P*-values < .05 were considered as CRC-related genes. Volcano plots and hazard ratio distribution plots were generated using ggplot2.

### 2.4. Network pharmacology analysis

Chemical components and targets of GQD were retrieved from the Traditional Chinese Medicine Systems Pharmacology Database and Analysis Platform, https://old.tcmsp-e.com/tcmsp.php). Bioactive compounds were selected based on oral bioavailability (≥ 20%) and drug-likeness (≥ 0.1). Target gene symbols were standardized using UniProt (https://www.uniprot.org/), and nonhuman targets were excluded. The intersection of GQD, T2DM, and CRC-related genes was determined as potential therapeutic targets of GQD. Kyoto Encyclopedia of Genes and Genomes and Gene Ontology enrichment analyses were conducted using the clusterProfiler package,^[13]^ with a significance threshold of *P* < .05. Networks were visualized and analyzed using Cytoscape 3.10.2 software.

### 2.5. Immunoinfiltration analysis

Immunoinfiltration assessment of the GSE115313 data set was performed using the IOBR package.^[14]^ The proportion of 22 immune cell types was estimated using the CIBERSORT algorithm. Differences in immune cell infiltration between cancerous and healthy tissues were compared, and the correlation between GQD’s potential targets and immunoinfiltration was analyzed.

### 2.6. Drug sensitivity assessment

Drug sensitivity was assessed using the pRRophetic package.^[15]^ Sensitivity to 45 drugs was evaluated in the GSE115313 cohort, and differences between cancerous and healthy tissues were compared. The correlation between GQD’s potential targets and drug sensitivity was analyzed.

### 2.7. Molecular docking

Protein structures of potential targets of GQD for the treatment of T2DM and CRC were obtained from the Protein Data Bank (https://www.rcsb.org/) using PyMOL 2.3.2 software. Hydrogens were added, and partial charges were computed using AutoDockTools 1.5.7. Docking grids were generated using the PyMOL plugin GetBox. Structures of bioactive compounds were downloaded from PubChem (https://pubchem.ncbi.nlm.nih.gov/). Molecular docking was performed using the Lamarckian genetic algorithm. Docking results were visualized using PyMOL and LigPlus.

## 3. Results

### 3.1. Identification of genes associated with CRC

**Figure 2.**
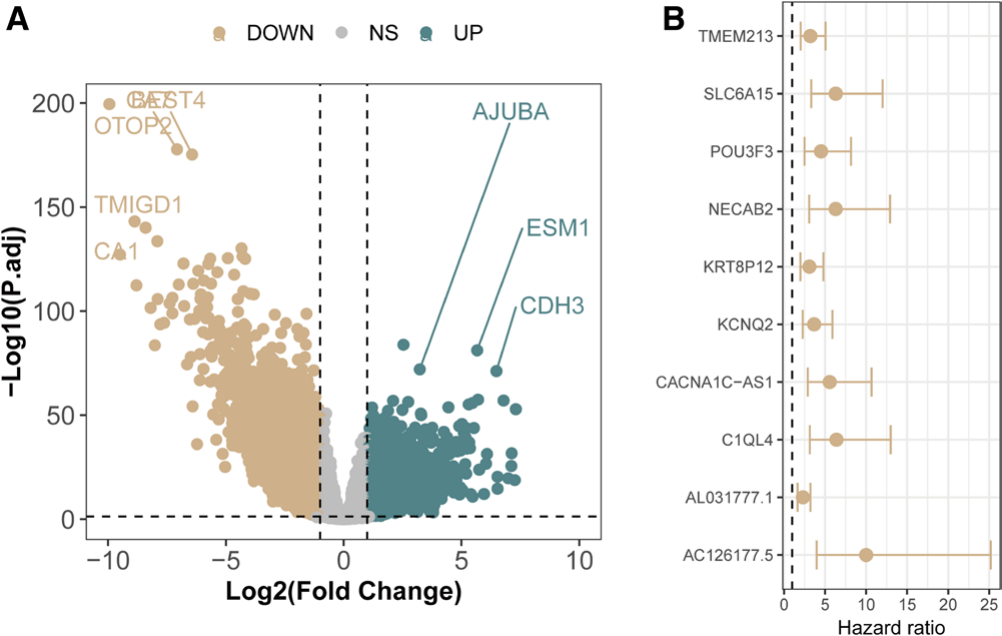
Identification of colorectal cancer-related genes. (A) Volcano plot of differential expression analysis for the TCGA-COAD cohort. (B) Heatmap of the top 10 prognosis-related genes in colorectal cancer. COAD = colorectal adenocarcinoma, TCGA = The Cancer Genome Atlas.

To identify genes associated with CRC, we first performed differential expression analysis. The results indicated that 7861 genes were aberrantly expressed in colorectal cancer tissues compared with normal tissues, with 3791 genes upregulated and 4070 genes downregulated (Fig. 2A). Further univariate Cox regression analysis revealed that 2644 of these differentially expressed genes were associated with CRC prognosis. Among these, 2367 genes were identified as risk genes, while the remaining 277 genes were classified as protective genes (Table S1, Supplemental Digital Content, https://links.lww.com/MD/P406). Figure 2B displays the top 10 CRC prognosis-related genes with the lowest *P*-values. Consequently, these 2644 genes were used for subsequent analyses to identify shared genes with T2DM.

### 3.2. Identification of genes associated with T2DM

**Figure 3.**
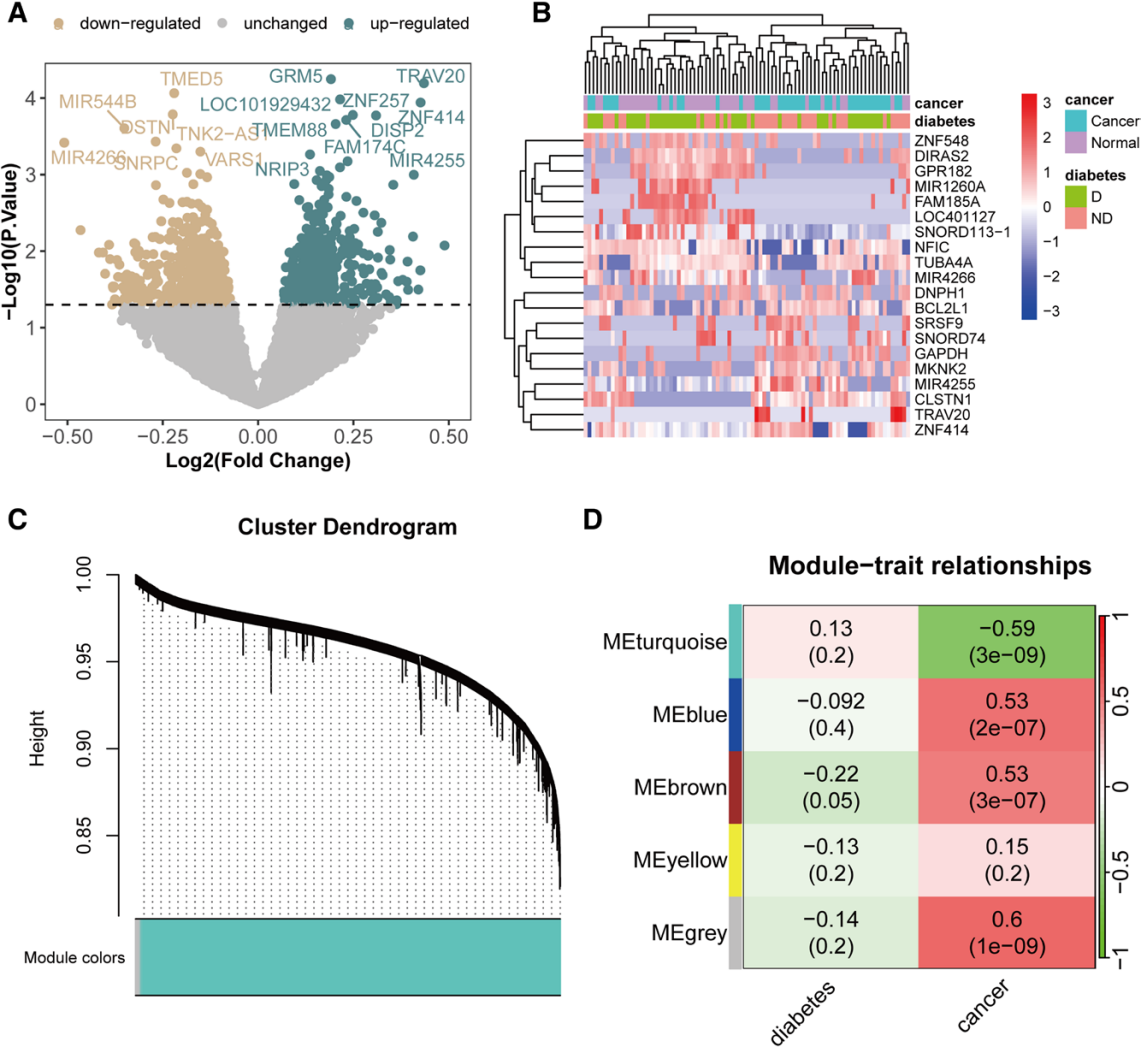
Identification of T2DM-related genes. (A) Volcano plot of differential expression analysis for the GSE115313 cohort comparing nondiabetic and diabetic patients. (B) Heatmap of differentially expressed genes. (C) Cluster dendrogram of modules based on the coexpression network. (D) Heatmap of the module-trait relationships. T2DM = type 2 diabetes mellitus.

To identify genes related to T2DM, we first conducted differential expression analysis on the GSE115313 cohort. The results indicated that 1048 genes were differentially expressed between diabetic and nondiabetic patients, with 549 genes overexpressed and 499 genes underexpressed (Fig. 3A). Figure 3B shows heatmaps of the 10 most highly and 10 most lowly expressed genes based on log(fold change). Further WGCNA identified 5 modules (turquoise, blue, brown, yellow, and gray; Fig. 3C). All modules except the yellow module were significantly associated with cancer, although none were significantly associated with diabetes (Fig. 3D). Therefore, modules with correlation coefficients >0.1 were selected for subsequent analysis.

### 3.3. Potential targets of GQD for the treatment of CRC+T2DM

**Figure 4.**
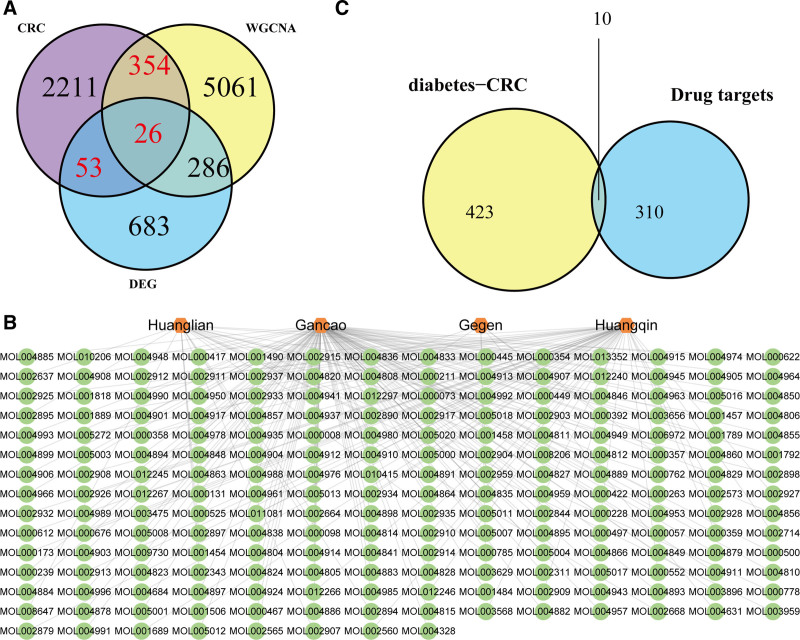
Identification of potential targets of GQD for the treatment of T2DM and CRC. (A) Venn diagram showing the overlap between T2DM and CRC-related genes. (B) Network of medicinal herbs and bioactive components in GQD. (C) Venn diagram identifying potential therapeutic targets of GQD for the treatment of T2DM and CRC. CRC = colorectal cancer, GQD = GeGen-QinLian decoction, T2DM = type 2 diabetes mellitus.

By combining the genes from WGCNA modules and differentially expressed genes, we identified 6463 T2DM-related genes (Fig. 4A). A Venn diagram demonstrated that there were 433 shared genes between these T2DM-related genes and the 2644 CRC-related genes (Fig. 4A). Enrichment analysis revealed that these genes were involved in several biological processes, including gene expression regulation, cell cycle control, apoptosis regulation, the Wnt signaling pathway, regulation of NF-κB transcription factor activity, and inflammatory mediator regulation of transient receptor potential (TRP) channels (Table S2, Supplemental Digital Content, https://links.lww.com/MD/P406). Through the Traditional Chinese Medicine Systems Pharmacology Database and Analysis Platform database, we identified 204 bioactive components from 4 Chinese medicinal herbs in GQD: Huangqin (56 components), Gegen (7 components), Gancao (125 components), and Huanglian (24 components) (Fig. 4B). Further analysis yielded 320 target proteins (Table S3, Supplemental Digital Content, https://links.lww.com/MD/P406). A Venn diagram identified 10 shared targets between the GQD targets and the genes related to T2DM and CRC (Fig. 4C): ADRA1B (adrenergic receptor alpha 1B), CALM1 (calmodulin 1), CDKN2A (cyclin-dependent kinase inhibitor 2A), CTNNA1 (cadherin-associated protein, alpha 1), FCER2 (Fc fragment of IgE receptor II), GSR (glutathione reductase), GSTM1 (glutathione S-transferase mu 1), IL13 (interleukin 13), INSR (insulin receptor), and MAPK9 (mitogen-activated protein kinase 9, also known as JNK2). These targets were considered potential therapeutic targets for GQD in the subsequent analysis.

### 3.4. Expression validation of potential targets for GQD in CRC+T2DM

**Figure 5.**
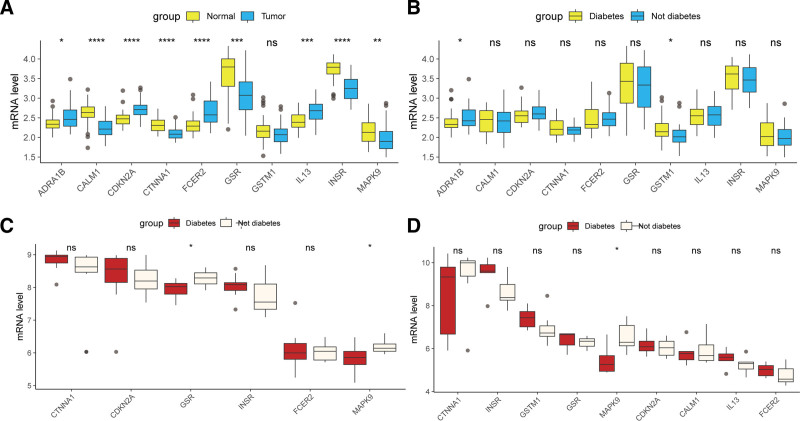
Expression characteristics of potential therapeutic targets of GQD for the treatment of T2DM and CRC. (A) Gene expression comparison in cancer vs normal tissues in the GSE115313 cohort. (B) Gene expression comparison in nondiabetic vs diabetic patients in the GSE115313 cohort. (C) Gene expression comparison in nondiabetic vs diabetic patients in the GSE20966 cohort. (D) Gene expression comparison in nondiabetic vs diabetic patients in the GSE25724 data set. ns, not significant, *P < .05, **P < .01, ***P < .001, ****P < .0001. CRC = colorectal cancer, GQD = GeGen-QinLian decoction, T2DM = type 2 diabetes mellitus.

To validate the expression of these potential targets in CRC and T2DM, we performed comparisons across multiple data sets. The results showed that, compared with normal tissues, the expression of ADRA1B, CDKN2A, FCER2, and IL13 was significantly upregulated in colorectal cancer tissues, while CALM1, CTNNA1, GSR, INSR, and MAPK9 were significantly downregulated. No significant difference observed for GSR (Fig. 5A). In addition, compared with nondiabetic patients, diabetic patients had significantly downregulated ADRA1B and upregulated GSTM1 in colorectal tissue (Fig. 5B). In the GSE20966 cohort, diabetes was associated with lower GSR and MAPK9 gene expression compared with nondiabetic controls (Fig. 5C). In the GSE25724 data set, diabetes was also associated with lower MAPK9 gene expression (Fig. 5D).

### 3.5. Enrichment analysis of potential targets for GQD in CRC+T2DM

**Figure 6.**
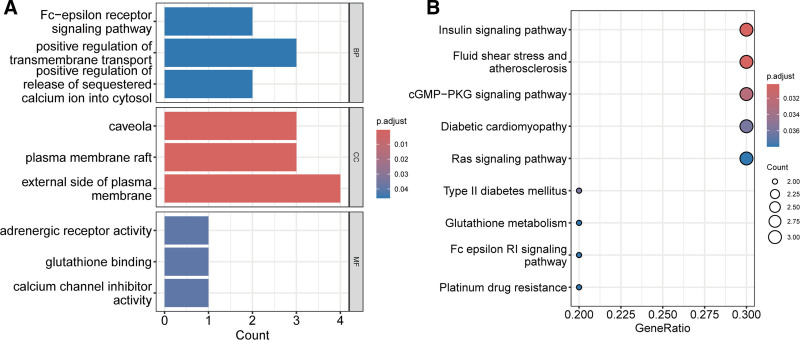
Enrichment analysis of potential targets of GQD for the treatment of T2DM and CRC. (A) GO enrichment analysis. (B) KEGG pathway enrichment analysis. CRC = colorectal cancer, GO = Gene Ontology, GQD = GeGen-QinLian decoction, KEGG = Kyoto Encyclopedia of Genes and Genomes, T2DM = type 2 diabetes mellitus.

We performed enrichment analysis on the 10 potential targets. The results showed that, in biological processes, these targets were associated with the Fc-epsilon receptor signaling pathway, positive regulation of transmembrane transport, and positive regulation of the release of sequestered calcium ions into the cytosol. In cellular components, the targets were associated with caveolae, plasma membrane rafts, and the external side of the plasma membrane. In molecular functions, these genes were linked to adrenergic receptor activity, glutathione binding, and calcium channel inhibitor activity (Fig. 6A). In Kyoto Encyclopedia of Genes and Genomes pathways, these genes were associated with the insulin signaling pathway, cyclic guanosine monophosphate (cGMP)-protein kinase G (PKG) signaling pathway, Ras signaling pathway, and Fc-epsilon receptor I signaling pathway (Fig. 6B).

### 3.6. Survival analysis of potential targets for GQD in CRC+T2DM

**Figure 7.**
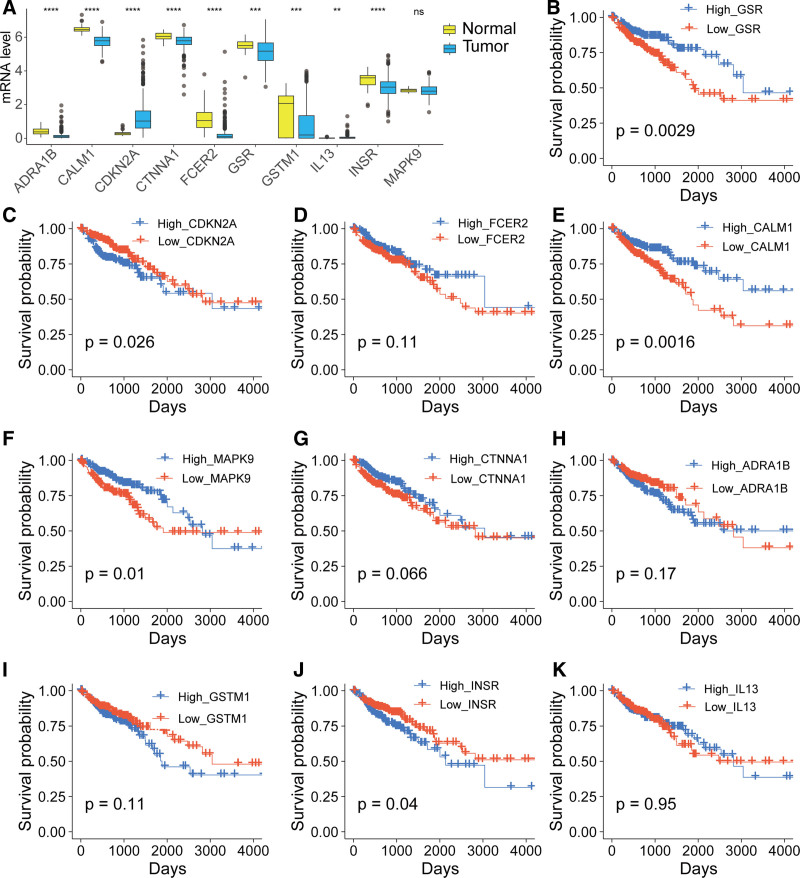
Expression and survival analysis of potential targets of GQD for the treatment of T2DM and CRC in the TCGA-COAD cohort. (A) Gene expression comparison. (B–K) Kaplan–Meier survival analysis. COAD = colorectal adenocarcinoma, CRC = colorectal cancer, GQD = GeGen-QinLian decoction, T2DM = type 2 diabetes mellitus, TCGA = The Cancer Genome Atlas.

In the TCGA-COAD cohort, we compared the expression of 10 potential genes in cancer and normal tissues. The results showed that, compared with normal tissues, the expression of ADRA1B, CALM1, CTNNA1, FCER2, GSR, GSTM1, and INSR was significantly downregulated, while CDKN2A and IL13 were significantly upregulated. No significant difference observed for MAPK9 (Fig. 7A). Kaplan–Meier survival analysis showed that high expression of GSR, CALM1, and MAPK9 was significantly associated with better prognosis in colorectal cancer patients. Conversely, high expression of CDKN2A and INSR was significantly associated with worse prognosis. No significant differences were observed for the other genes (Fig. 7B–7K).

### 3.7. Correlation of potential targets for GQD in CRC + T2DM with immune infiltration and drug sensitivity

**Figure 8.**
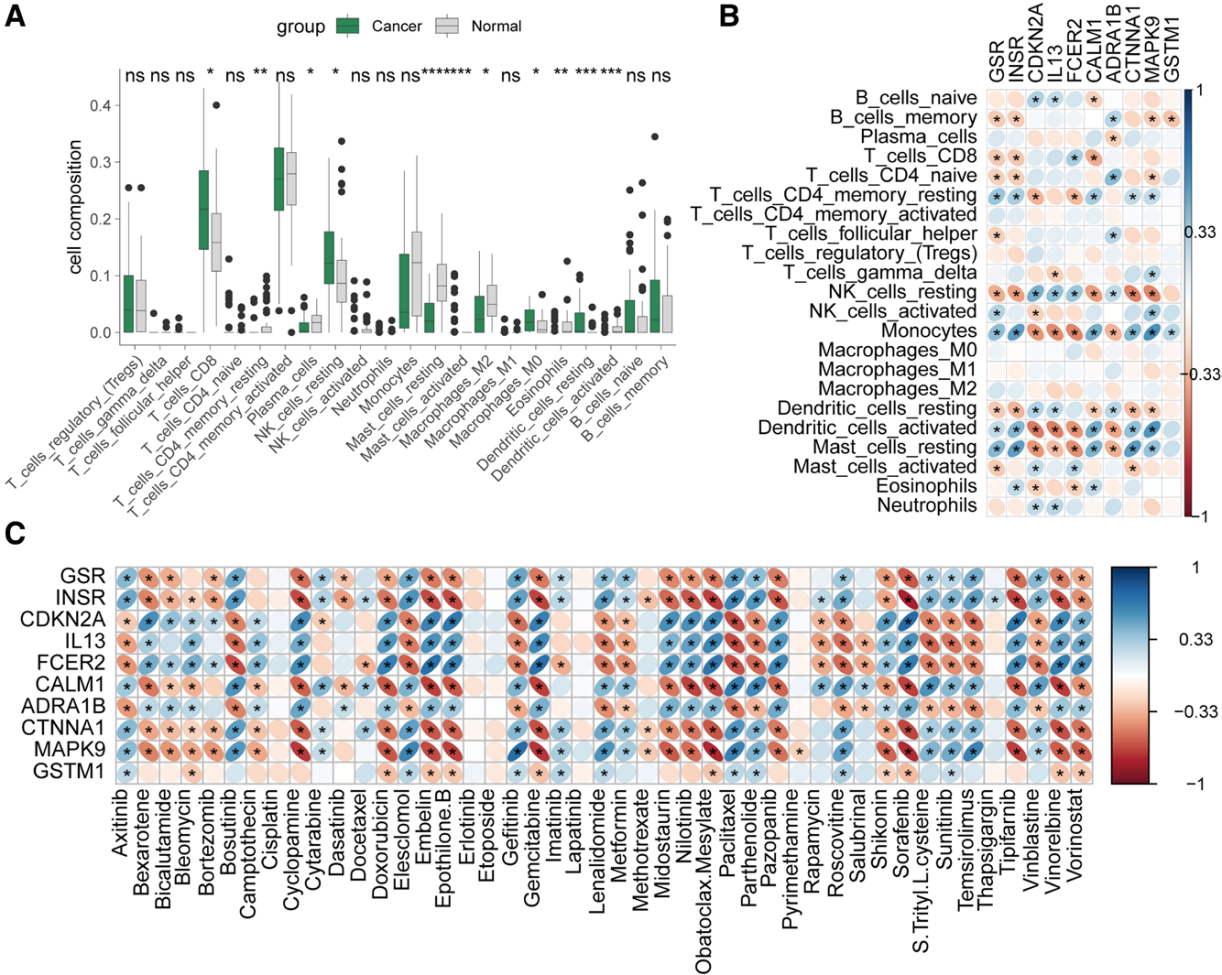
Correlation of potential targets of GQD for the treatment of T2DM and CRC with immune infiltration and drug sensitivity. (A) Comparison of immune cell infiltration in cancer vs normal tissues in the GSE115313 cohort. (B) Heatmap of the correlation between the 10 potential targets and immune cell infiltration. (C) Heatmap of the correlation between the 10 potential targets and drug sensitivity. CRC = colorectal cancer, GQD = GeGen-QinLian decoction, T2DM = type 2 diabetes mellitus.

We evaluated immune cell infiltration in the GSE115313 cohort and found that, compared with normal tissues, cancer tissues showed significantly increased infiltration of CD8 + cells, resting NK cells, M0 macrophages, and resting dendritic cells. Conversely, infiltration of resting CD4 + memory T cells, plasma cells, resting mast cells, M2 macrophages, eosinophils, and activated dendritic cells was significantly reduced (Fig. 8A). The 10 potential targets were found to be significantly correlated with infiltration of B cells, CD4+, CD8+, NK cells, dendritic cells, mast cells, eosinophils, and neutrophils (Fig. 8B). Drug sensitivity analysis showed that these targets were associated with sensitivity to various drugs, including axitinib, paclitaxel, docetaxel, and gefitinib. Specifically, consistent correlations were observed for CDKN2A, IL13, FCER2, and ADRA1B, while the remaining genes showed consistent correlations in the opposite direction (Fig. 8C).

### 3.8. Affinity analysis of potential targets for GQD in CRC + T2DM and their bioactive components

**Figure 9.**
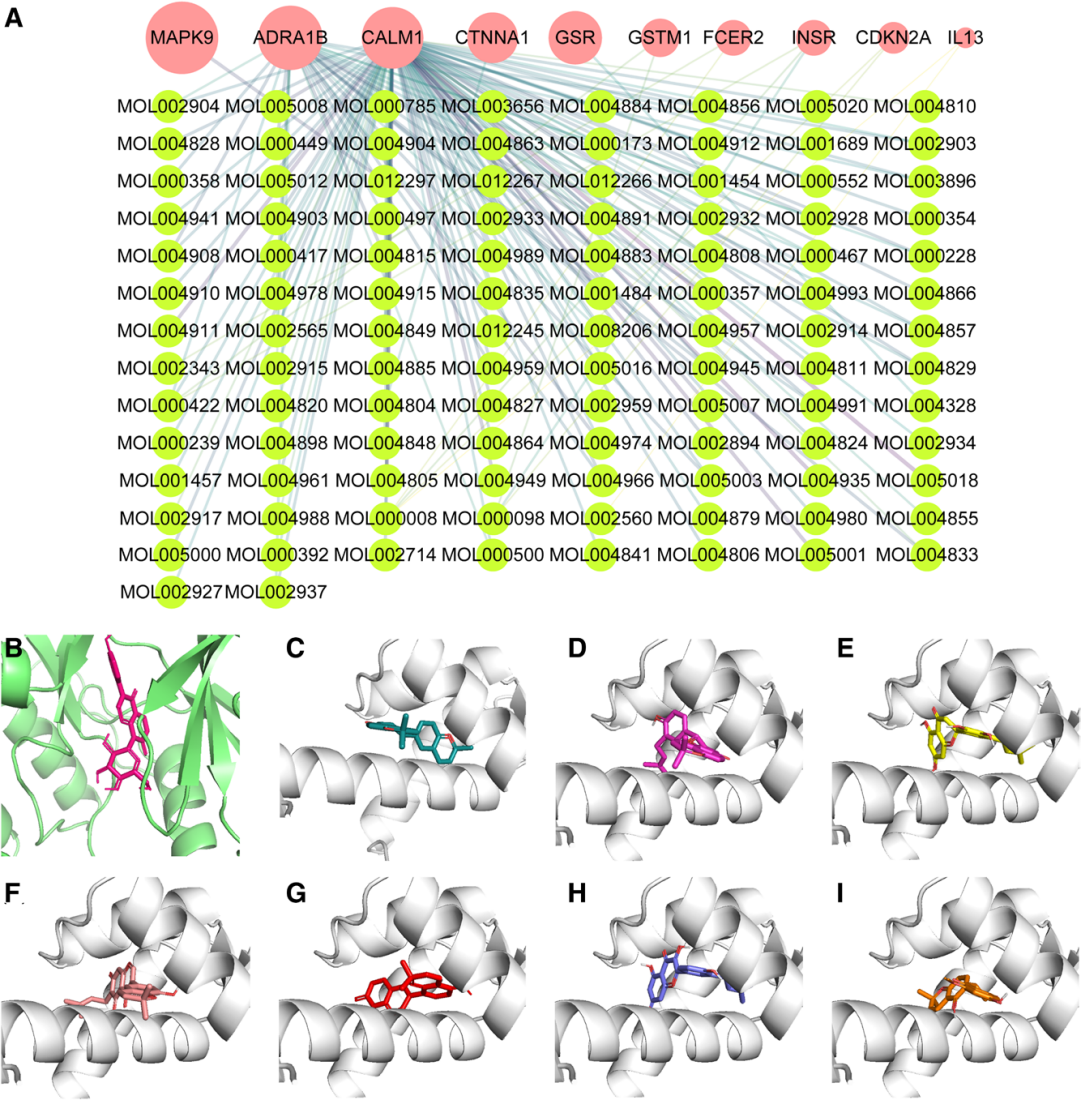
Molecular docking of potential targets of GQD for the treatment of T2DM and CRC with their bioactive components. (A) Network of 10 potential targets and bioactive components. (B) 3D structure of the MAPK9-puerarin complex, (C) CALM1-xambioona complex, (D) CALM1-shinflavanone complex, (E) CALM1-sigmoidin-B complex, (F) CALM1-gancaonin H complex, (G) CALM1-glabrene complex, (H) CALM1-isolicoflavonol complex, (I) CALM1-glyasperins M complex. CALM1 = calmodulin 1, CRC = colorectal cancer, GQD = GeGen-QinLian decoction, MAPK9 = mitogen-activated protein kinase 9, T2DM = type 2 diabetes mellitus.

To assess the binding affinity between these potential targets and bioactive components, we performed molecular docking analysis. We constructed a network of potential targets and bioactive components, identifying 106 bioactive components for the 10 targets and 144 interactions (Fig. 9A). Docking results showed that all bioactive components had binding energies less than −5 kcal/mol, indicating good binding capabilities (Table S4, Supplemental Digital Content, https://links.lww.com/MD/P406). The 8 components with the lowest binding energies were CALM1-xambioona (−10.6 kcal/mol), CALM1-shinflavanone (−10.5 kcal/mol), CALM1-sigmoidin-B (−10.3 kcal/mol), CALM1-gancaonin H (−10 kcal/mol), CALM1-glabrene (−9.9 kcal/mol), CALM1-isolicoflavonol (−9.8 kcal/mol), CALM1-glyasperins M (−9.6 kcal/mol), and MAPK9-puerarin (−9.6 kcal/mol). These interactions are illustrated in Figure 9B–9I and Figure S1, Supplemental Digital Content, https://links.lww.com/MD/P407.

## 4. Discussion

Cotreatment of different diseases holds significant theoretical and clinical significance, and the use of TCM for concurrent diseases has been widely successful. Western medicine often employs metformin in the treatment of CRC complicated with T2DM, achieving reliable efficacy.^[16]^ Long-term administration of antidiabetic drugs can improve the prognosis of patients after CRC surgery and prolong their survival.^[17]^ Studies have found that T2DM and CRC are closely related in terms of pathogenesis, diagnosis, treatment and other aspects, and T2DM can affect the early screening of CRC.^[18–20]^ According to TCM theory, CRC and T2DM share partially overlapping pathogenic mechanisms, such as spleen and stomach dysfunction. Therefore, the potential value of TCM in the combined treatment of these 2 diseases has been proposed. Clinically, GQD has been confirmed to be effective for both T2DM and CRC, respectively.^[6,7,10]^ Inspired by these findings, we sought to elucidate the common molecular features involved in the pathogenesis of CRC and T2DM and reveal the pharmacological basis of GQD for treating these 2 diseases. To achieve this, we integrated bioinfomatics, network pharmacology and molecular docking.

In this study, we identified 433 genes shared between CRC and T2DM, which participate in biological processes crucial for both conditions. The Wnt signaling pathway is a key target pathway for metabolic disorders, with many components of Wnt signaling involved in glucose homeostasis.^[21]^ Studies have shown that Wnt signaling mediates the Toll-like receptor pathway, promoting uncontrolled lipogenesis and inflammation, making it a potential therapeutic target for obesity and T2DM.^[22]^ The Wnt pathway is also closely linked to various aspects of cancer biology, including proliferation, stemness, apoptosis, autophagy, metabolism, inflammation, immunity, microenvironment, drug resistance, ion channels, heterogeneity, epithelial-mesenchymal transition, migration, invasion, and metastasis.^[23]^ Drugs, phytochemicals, and molecular formulations targeting the Wnt pathway for the treatment of colorectal cancer have been developed,^[24]^ and Wnt inhibitors are clinically utilized for colorectal cancer therapy.^[25–27]^ Constitutive activation of NF-κB occurs in 40% to 80% of colorectal cancers, and its involvement in colorectal carcinogenesis likely includes activation of antiapoptotic gene expression, enhancement of cell survival and proliferation, regulation of angiogenesis, and promotion of cancer cell metastasis.^[28]^ Chronic low-grade inflammation is considered part of the pathogenesis of T2DM.^[29]^ NF-κB plays a role in this inflammatory process by regulating the production of pro-inflammatory cytokines such as TNF-α and IL-1β.^[30]^ Therefore, drugs targeting NF-κB are potential candidates for the treatment of both T2DM and CRC.^[31]^ The TRP superfamily consists of a variety of nonselective cation channels with widespread tissue distribution, participating in numerous physiological processes, including sensory perception, hormone secretion, vasoconstriction/vasodilation, and cell cycle regulation.^[32]^ Several TRP channels have been shown to play roles in regulating hormone release, energy expenditure, pancreatic function, and the release of neurotransmitters controlling obesity and/or diabetes.^[33]^ Moreover, dietary supplementation with natural ligands of TRP channels has been shown to have potential beneficial effects on obesity and diabetes.^[33]^ Simultaneously, TRPs are implicated in the development of colorectal cancer^[34]^ and can serve as predictive markers and potential indicators of chemoresistance in colorectal cancer.^[35]^

TCM has accumulated extensive experience in the properties of various Chinese medicinal materials over a long period and has combined them into a variety of effective prescriptions through symptomatic treatment strategies. Modern pharmacological studies have further confirmed the activity of GQD herbs in CRC complicated with T2DM. For instance, *Scutellaria baicalensis* has been shown to improve the therapeutic effects of CRC and reverse radio-resistance in different cancers.^[36]^ Hydrophobic flavonoids from *S. baicalensis* induced CRC cell apoptosis through a mitochondrial-mediated pathway.^[37]^ Meanwhile, the active compounds in *S. baicalensis* have been shown to improve the renal function, insulin resistance and retinopathy of patients with T2DM.^[38]^ However, the mechanism by which these herbs coordinate the treatment of T2D and CRC in appropriate proportions is not fully understood and requires further molecular analysis. In this study, network pharmacology provided details of the active ingredients in these herbs against T2DM and CRC. Topological analysis revealed several active ingredients with pivotal roles, including quercetin, apigenin, formononetin, etc. Quercetin is a well-known polyphenol with antidiabetes and anticancer activities, highlighting its importance in the combined treatment of 2 diseases. Apigenin ameliorated diabetic nephropathy by decreasing mitochondrial oxidative stress and various pathways,^[39]^ and similar targets or pathways also mediated the inhibitory effect of apigenin on CRC.^[40]^ Despite these pharmacological advances, investigation of the synergistic effects of active ingredients presents a greater challenge and requires a wider range of technologies, such as transcriptomics and metabolomics.

This study identified ten potential targets for the treatment of T2DM combined with CRC using GQD, among which GSR and MAPK9 were aberrantly expressed in both T2DM and CRC and significantly associated with CRC prognosis. GSR is essential for the glutathione/glutaredoxin antioxidant system, catalyzing the reduction of oxidized glutathione disulfide back to its reduced form, which in turn reduces oxidized glutaredoxin.^[41]^ Recent studies have shown that the immunohistochemical expression of GSR correlated significantly with tumor histological grade, depth of invasion, regional lymph node involvement, tumor stage, and proliferating cell nuclear antigen immunohistochemical expression, suggesting that GSR immunohistochemical expression may serve as an independent prognostic factor in patients with colorectal adenocarcinoma.^[42]^ High blood glucose levels can lead to excessive production of free radicals within cells, damaging cellular structures and functions. Thus, the cellular antioxidant system involving GSR may be involved in the onset and progression of diabetes. Studies have demonstrated that polymorphisms in the GSR gene were associated with susceptibility to T2DM.^[43]^ The JNK (c-Jun N-terminal kinase) group of MAPKs is associated with T2DM because these protein kinases can phosphorylate IRS1 (insulin receptor substrates), thereby inhibiting insulin signaling.^[44,45]^ In the peripheral blood leukocytes of patients with CRC and diabetes, MAPK9 expression was abnormally reduced.^[46]^ In colon cancer, MAPK9-mediated reactive oxygen species-induced autophagy, and targeted intervention of MAPK9 expression can inhibit autophagy.^[47]^ Consequently, GSR and MAPK9 are potential therapeutic targets for T2DM and CRC comorbidity.

Enrichment analysis revealed that these targets participate in multiple signaling pathways. The insulin signaling pathway activated downstream PI3K-AKT pathways, regulating processes such as glucose uptake, glycogen synthesis, and fatty acid synthesis.^[48]^ Persistent activation of this pathway can stimulate the proliferation of colon cancer cells^[49]^ and inhibit apoptosis.^[50]^ The cGMP-PKG signaling pathway may influence the function of pancreatic beta cells, including insulin secretion and cell survival.^[51]^ PKG, the central effector of cGMP, can inhibit tumor proliferation and angiogenesis through suppression of β-catenin/TCF and SOX9 signaling. Therapeutic activation of the cGMP/PKG pathway offers a promising approach for the prevention and treatment of colon cancer.^[52]^ Ras proteins activated multiple downstream signaling pathways, including the Raf-1 proto-oncogene, serine/threonine kinase/mitogen-activated protein kinase kinase/extracellular signal-regulated kinases pathway and the PI3K/Akt pathway. Aberrant activation of these pathways can promote cell proliferation and survival and mediated insulin resistance.^[53–55]^

Despite the promising findings, several limitations in this study should be acknowledged. While network pharmacology can explore pharmacodynamic components and related mechanisms by integrating drug targets and human disease targets, it is not comprehensive for complex human systems. Notably, the regulation of TCM on the intestinal flora is an important aspect. Studies have shown that gut microbiota was inextricably related to human diseases and was involved in the pathogenesis of diseases. However, current network pharmacology technology make it difficult to elucidate the effect of TCM on gut microbiota in disease treatment and its relationship with efficacy. Additionally, further experimental verification is needed to confirm the findings of this study.

## 5. Conclusion

In summary, our study identified 433 shared genes between T2DM and CRC, which are involved in gene expression regulation, cell cycle control, apoptosis regulation, Wnt signaling pathway, regulation of NF-κB transcription factor activity, and inflammatory mediator regulation of TRP channels. Network pharmacology further pinpointed ten potential targets for the treatment of T2DM combined with CRC, with these genes participating in the insulin signaling pathway, cGMP-PKG signaling pathway, Ras signaling pathway, and Fc-epsilon receptor I signaling pathway. Molecular docking confirmed the affinity of these ten targets with the active components of GQD. Further experimental validation is warranted to explore the biological functions of these genes in the context of T2DM and CRC.

## Author contributions

**Conceptualization:** Jinhao Liang, Chengjiang Xiang, Yuanxiao Liang.

**Data curation:** Jinhao Liang, Chengjiang Xiang, Yuanxiao Liang.

**Formal analysis:** Jinhao Liang, Chengjiang Xiang, Yuanxiao Liang.

**Funding acquisition:** Jinhao Liang, Chengjiang Xiang, Yuanxiao Liang.

**Investigation:** Jinhao Liang, Chengjiang Xiang, Yuanxiao Liang.

**Methodology:** Jinhao Liang, Chengjiang Xiang, Yuanxiao Liang.

**Project administration:** Jinhao Liang, Chengjiang Xiang, Yuanxiao Liang.

**Resources:** Jinhao Liang, Chengjiang Xiang, Yuanxiao Liang.

**Software:** Jinhao Liang, Chengjiang Xiang, Yuanxiao Liang.

**Supervision:** Jinhao Liang, Chengjiang Xiang, Yuanxiao Liang.

**Validation:** Jinhao Liang, Chengjiang Xiang, Yuanxiao Liang.

**Visualization:** Jinhao Liang, Chengjiang Xiang, Yuanxiao Liang.

**Writing – original draft:** Jinhao Liang, Chengjiang Xiang, Yuanxiao Liang.

**Writing – review & editing:** Jinhao Liang, Chengjiang Xiang, Yuanxiao Liang.

## Supplementary Material


