# Supplementary material for: Network pharmacology and bioinformatics insight into the mechanism of GeGen-QinLian decoction in colorectal cancer and type 2 diabetes mellitus
Source: Medicine (Baltimore). 2025 Jul 18;104(29):e43274. doi: 10.1097/MD.0000000000043274 (PMC12282716; doi:10.1097/MD.0000000000043274)

**Figure S1.** The 2D structure of the (A) MAPK9-puerarin complex, (B) CALM1-xambioona complex, (C) CALM1-shinflavanone complex, (D) CALM1-sigmoidin-B complex, (E) CALM1-gancaonin H complex, (F) CALM1-glabrene complex, (G) CALM1-isolicoflavonol complex, (H) CALM1-glyasperins M complex.


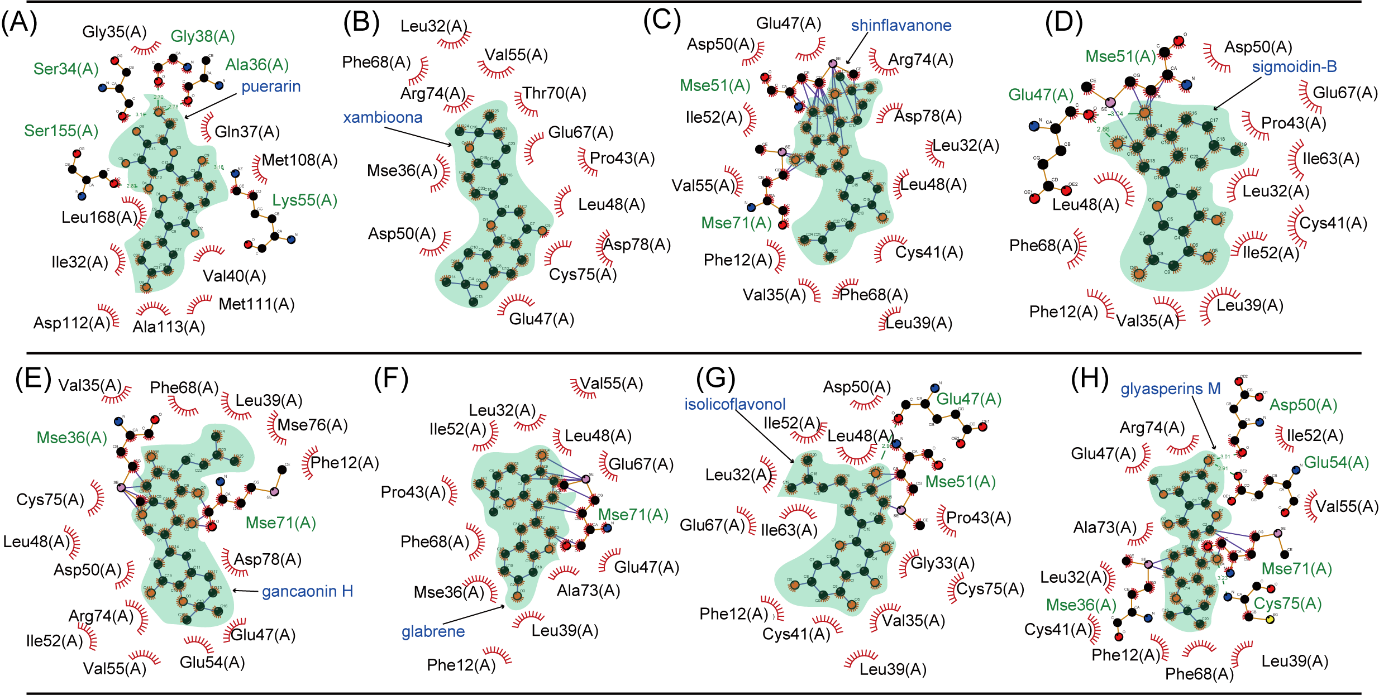

Supplement: Supplementary file 2 [file medi-104-e43274-s002.docx]
